# Microplastic Incorporation into Soil in Agroecosystems

**DOI:** 10.3389/fpls.2017.01805

**Published:** 2017-10-18

**Authors:** Matthias C. Rillig, Rosolino Ingraffia, Anderson A. de Souza Machado

**Affiliations:** ^1^Institut für Biologie, Plant Ecology, Freie Universität Berlin, Berlin, Germany; ^2^Berlin-Brandenburg Institute of Advanced Biodiversity Research, Berlin, Germany; ^3^Department of Agricultural, Food and Forestry Sciences, Università di Palermo, Palermo, Italy

**Keywords:** microplastic, nanoplastic, agroecosystem, soil biota, tillage, soil aggregation, porosity, contaminant transport

## Background

We live in a plastic age (Thompson et al., 2009), with microplastic (typically defined as plastic particles < 5 mm) becoming an increasingly appreciated aspect of environmental pollution. Research has been overwhelmingly focused on aquatic systems, especially the oceans, but there is a current shift to more strongly consider terrestrial ecosystems (Rillig, 2012; Horton et al., 2017). In particular agroecosystems are coming into focus as a major entry point for microplastics in continental systems (Nizzetto et al., 2016b), where contamination might occur via different sources as sludge amendment or plastic mulching (Steinmetz et al., 2016). Given the central role of agroecosystems, including their soil biodiversity (Rillig et al., 2016), in food production, such numbers are potential cause for concern. Field data on measured microplastic presence in agricultural soils are still not widely available, but nevertheless this material is certain to arrive at the soil surface. The fate of material deposited at the soil surface is not clear: particles may be removed by wind or water erosion, becoming airborne, or may be lost by surface runoff (Nizzetto et al., 2016a). Nevertheless, a substantial part of the microplastic (or nanoplastic following further disintegration) is expected to enter the soil.

The degree of hazard represented by microplastic to various soil biota is not clear. Direct evidence comes from experimental work on earthworms, on which microbeads had negative effects (Huerta Lwanga et al., 2016; also reviewed in Horton et al., 2017). Data on impacts on other soil biota groups are not available. However, Kiyama et al. (2012) have shown that polystyrene beads can be taken up by the nematode *Caenorhabditis elegans*; this means the material could also accumulate in the soil food web (Rillig, 2012). Movement into soil is an important aspect of assessing risk: will soil biota be exposed to microplastics? Here, we sketch what is known about movement of such particles in soil, which players and factors could influence this, and we chart avenues for research aimed at the movement and distribution of microplastic in agricultural soils.

## Evidence of microplastic transport in soil

Early evidence for transport of microplastic particles comes from work in which researchers have used plastic particles as tracers to monitor particle movement through porous media from a soil physical perspective. This is a research topic on which abundant experimental data from column studies exist, and also conceptual understanding in the form of mathematical models describing physical features (McDowell-Boyer et al., 1986). For example, studies using mostly packed sand have used polystyrene latex particles (0.2 μm) of different hydrophobicity (Wan and Wilson, 1994), 0.468-μm latex microspheres (Roy and Dzombak, 1997), or latex particles of 0.11 μm size (Grolimund et al., 1998). Since these studies were typically aimed at colloidal behavior (as transport agents of pollutants), the particle sizes are smaller than the ones typically examined for microplastic in environmental assessments; this notwithstanding, there is clear evidence that such particles move through a packed sand or soil (Grolimund et al., 1998) column in the lab, and that retention also depends on particle hydrophobicity (Wan and Wilson, 1994). In the field, microplastic fibers were present near preferential flow paths and below the soil mixing layer, suggesting the surface-applied fibers (in sludge) had also moved (over a period of 15 year) (Zubris and Richards, 2005).

More recently, the action of soil biota, specifically animals, has been examined as a driver of microplastic incorporation into soil. For example, Huerta Lwanga et al. (2016) showed size-specific incorporation and concentration of microplastic beads in earthworm casts, Huerta Lwanga et al. (2017) have shown that earthworms can incorporate microplastic beads into their burrows from the surface, and Rillig et al. (2017) showed that earthworms can enhance the movement of microplastic beads down the soil profile from the surface in the laboratory. Smaller microbeads moved more readily down the soil profile in this experiment.

In summary, both in terms of the more physical aspects and for soil biota there is now experimental evidence that microplastic particles are moved into the soil when deposited at the soil surface. This clearly establishes a case for exposure of soil biota, including roots and the soil and rhizosphere microbiome, to these particles. It is now necessary to explore the various factors that may affect transport of these particles.

## Features affecting microplastic movement into and within soil

Several soil features, soil biota activities or management actions can potentially influence the movement of microplastic into and within the soil. Biopores (macropores created by soil biota), and plowing, as well as soil cracking are likely most responsible for downward movement, whereas soil biota (mesofauna, fungi), harvesting, and plowing would serve to distribute the particles also horizontally. Transport will additionally be influenced by particle properties, and also by processes potentially sequestering them, such as soil aggregation.

### Microplastic particle properties

Size, hydrophobicity, charge, density, and shape (fiber, bead, planar structure, foam) would all be expected to significantly influence movement of particles, with direct evidence existing for size (Rillig et al., 2017) and hydrophobicity (Wan and Wilson, 1994). Shape is particularly interesting since microbeads, so far used in experiments, will behave differently from microfibers. The latter are more likely to become entangled in the soil matrix and would be expected to interact differently with soil biota. Even though so far studied mostly in aquatic systems, microplastic particles in soil will likely also be surrounded by an ecocorona (Galloway et al., 2017), consisting of microbes and various organic deposits. This ecocorona could substantially influence the shape, size, and surface properties of particles, and therefore also their movement. Degradation of microplastic particles (e.g., photolysis at the soil surface, or biodegradation) could also influence movement by altering surface properties and the particle ecocorona.

### Macropores

Many physical factors affect the movement of particles through the soil, including physical attachment and detachment, sedimentation, and sieving. Macropores (pores > 0.08 mm) generally enhance movement of particles, because sedimentation and sieving are not as pronounced, and they enhance the movement of water. This means that all players affecting the presence of macropores will indirectly influence the efficiency with which microplastic particles are moved in soil. The most important producers of biopores, macropores of typically tubular shape, are earthworms and roots. In addition, soil aggregation, a joint physiochemical/ biotic process, leaves macropores in between structural units, the aggregates. Experimental data for earthworms already exist (e.g., Rillig et al., 2017), even though in these studies active earthworms were present, and it is therefore not clear what percentage of microplastic particles moved through existing earthworm biopores rather than with the animals. There are no data on roots, however. Especially in agricultural systems, after harvest, this could be a massive transport pathway as roots decompose, leaving biopores. Root systems differ widely, for example in terms of depth and also in terms of fineness. One could expect that deeply rooted plants with coarser roots may be most effective at facilitating the movement of particles.

### Soil cracking and wet-dry cycles

In agricultural soils with expanding mineral types, e.g., montmorillonite, cracks, and fissures can appear when soil dries. These cracks are open entryways for particles, that in this way could potentially move to substantial depths, very quickly arriving at deeper soil layers. Wet-dry cycles have been experimentally shown to directly mobilize colloid-sized particles in soils at a smaller scale (Majdalani et al., 2008), an effect the authors attributed to soil matrix weakening; similar patterns likely also hold for freeze-thaw cycles.

### Sequestration inside soil aggregates

Microplastic particles will likely become embedded inside of soil aggregates, even though the extent to which this happens is unknown. Soil aggregation is a dynamic process, with aggregates being formed and disintegrating. During formation of macroaggregates in hierarchically structured soils, microplastic particles, and microaggregates (< 0.250 mm) will be included along with organic matter, microbes, and primary soil particles (Tisdall and Oades, 1982). During the persistence of macroaggregates, which can range range to weeks and months (Peng et al., 2017), the microplastic particles contained therein would be retained in the soil profile.

### Soil biota

Other than as producers of biopores, soil biota can actively contribute to the movement of microplastic particles. Recently, microarthropods (collembola) have been shown to be able to move microplastic beads in a laboratory arena (Maaß et al., 2017). Such active, incidental, relatively small-scale transport could spread microplastic particles also horizontally, which may facilitate their subsequent entry into the soil. Similar effects can also be expected for mites, even though there is no experimental evidence yet. Perhaps fungal hyphae may also serve as preferential paths for movement of particles in the cm-range, as has been demonstrated for the transport of bacterial cells (Wick et al., 2007). The general literature on particle transport in soil by bioturbation (Gabet et al., 2003) also suggests that plant processes (e.g., root growth, uprooting) and various animals (earthworms, various larvae, vertebrates) can contribute to particle movement.

### Plowing and harvesting

In agroecosystems, plowing is a widespread practice, and through this activity microplastic particles can be very effectively moved into the soil to the depth of the plow. Different tillage practices affect different soil layers and thus the depth to which microplastic can be incorporated. For instance, conventional tillage practices affect usually the first 20–30 cm, while in no-tillage soil disturbance, to place the seeds, affects only the very top soil layer, generally a few centimeters (Paustian et al., 1997). In addition, under conventional tillage different types of plowing may differ in the extent to which they facilitate microplastic incorporation along the layer affected by the machinery. Moaldboard plowing brings about an inversion of the respective soil layer, with the consequence that microplastic present at the soil surface will mostly be brought to a single layer at the plowing depth. By contrast, other tillage practices such as shallow hallowing or harrowing, have a mixing effect, likely resulting in the distribution of the microplastic particles throughout the tillage layer.

Harvesting especially of plant portions below the soil surface (e.g., potatoes, carrots) can also serve to incorporate microplastic, albeit to a shallower depth, depending on the crop.

## Research needs and conclusions

There are clear research needs that emerge from the discussion above:

- Dedicated column experiments in the laboratory, and eventually in the field, will be necessary to estimate rates of movement of microplastic particles, and to disentangle the relative roles of the various factors potentially influencing movement. This should include an assessment of risk for microplastics reaching groundwater.
- Such experiments and other studies should not only include beads or approximately spherical particles but also fibers and other plastic particles. There is very little we know about the behavior of microplastic fibers in soil, despite their likely prominence (e.g., Zubris and Richards, 2005; Hartline et al., 2016; Hernandez et al., 2017).
- Interactions with soil aggregates should be a focus, because microplastic particles could be incorporated into soil aggregates, thereby immobilizing these particles. However, this also likely protects microplastics from microbial breakdown, increasing overall residence time; and given the aggregate dynamics, particles would be continuously re-released. Additionally, it is unclear what effects microplastics have on the soil aggregation process itself, which could affect macropores and ultimately particle movement.

Such future work is important: particles remaining at the surface could be moved around the landscape with potentially undesirable effects, but particles in the soil could have mostly unknown effects on soil biota and crop plants, possibly affecting food security. And, when microplastic particles move further through the soil profile, they would eventually also end up in groundwater. The contamination of subterranean waters with microplastic is of particular concern because they could have direct implications for human and animal health. Additionally, as a consequence of abrasion, chemical, or biodegradation occurring during transport, nanoplastic particles could be produced, posing fundamentally different hazards.

Many aspects discussed here also pertain to soils in other terrestrial ecosystems; however, it is evident that the specific combination of machinery-driven soil preparation, crop cultivation, and harvest dynamics, and unique microplastic exposure pathways make agricultural soils particularly vulnerable and important to study.

## Author contributions

MR: wrote the first draft of the paper; RI and AM: contributed ideas and text.

### Conflict of interest statement

The authors declare that the research was conducted in the absence of any commercial or financial relationships that could be construed as a potential conflict of interest.
